# Enhancing flavonoid production by promiscuous activity of prenyltransferase, BrPT2 from *Boesenbergia rotunda*

**DOI:** 10.7717/peerj.9094

**Published:** 2020-05-01

**Authors:** Yvonne Jing Mei Liew, Yean Kee Lee, Norzulaani Khalid, Noorsaadah Abd Rahman, Boon Chin Tan

**Affiliations:** 1 Institute of Biological Sciences, Faculty of Science, University of Malaya, Kuala Lumpur, Malaysia; 2 Department of Chemistry, Faculty of Science, University of Malaya, Kuala Lumpur, Malaysia; 3 Center for Research in Biotechnology for Agriculture, University of Malaya, Kuala Lumpur, Malaysia

**Keywords:** Flavonoids, Prenyltransferase, Ginger, Enzyme, Metabolites

## Abstract

Flavonoids and prenylated flavonoids are active components in medicinal plant extracts which exhibit beneficial effects on human health. Prenylated flavonoids consist of a flavonoid core with a prenyl group attached to it. This prenylation process is catalyzed by prenyltranferases (PTs). At present, only a few flavonoid-related *PT* genes have been identified. In this study, we aimed to investigate the roles of *PT* in flavonoid production. We isolated a putative *PT* gene (designated as *BrPT2*) from a medicinal ginger, *Boesenbergia rotunda*. The deduced protein sequence shared highest gene sequence homology (81%) with the predicted homogentisate phytyltransferase 2 chloroplastic isoform X1 from *Musa acuminata* subsp. *Malaccensis*. We then cloned the *BrPT2* into pRI vector and expressed in *B. rotunda* cell suspension cultures via *Agrobacterium*-mediated transformation. The *BrPT2*-expressing cells were fed with substrate, pinostrobin chalcone, and their products were analyzed by liquid chromatography mass spectrometry. We found that the amount of flavonoids, namely alpinetin, pinostrobin, naringenin and pinocembrin, in *BrPT2*-expressing cells was higher than those obtained from the wild type cells. However, we were unable to detect any targeted prenylated flavonoids. Further in-vitro assay revealed that the reaction containing the BrPT2 protein produced the highest accumulation of pinostrobin from the substrate pinostrobin chalcone compared to the reaction without BrPT2 protein, suggesting that BrPT2 was able to accelerate the enzymatic reaction. The finding of this study implied that the isolated *BrPT2* may not be involved in the prenylation of pinostrobin chalcone but resulted in high yield and production of other flavonoids, which is likely related to enzyme promiscuous activities.

## Introduction

Flavonoids, including flavanones and chalcones, are major constituents in many plant species. They are synthesized through phenylpropanoid pathway, starting with aromatic amino acid phenylalanine which is derived from the shikimate pathway. Flavonoids have a wide range of biological functions (Gill & Tuteja, 2010; Hassan & Mathesius, 2012; Mierziak, Kostyn & Kulma, 2014) and biochemical activities (Fathima & Rao, 2016) in plants. For instance, Tewtrakul et al. (2003) reported that pinostrobin, pinocembrin, cardamonin and alpinetin isolated from a medicinal ginger *Boesenbergia pandurata* (syn. *Boesenbergia rotunda*), exhibited considerable activity against HIV protease (Tewtrakul et al., 2003). Other compounds, such as panduratin A and 4′-hydroxypanduratin A, from this ginger exhibited inhibitory activity against dengue NS2B/3 serine protease which is mandatory for viral replication process (Tan et al., 2006). Recently, Youn & Jun (2019) demonstrated that numerous flavonoids (cardamonin, pinocembrin and pinostrobin) found in *B. rotunda* could be an alternative preventive agent for Alzheimar’s disease. The authors found that these flavonoids could inhibit the Beta-site amyloid precursor protein cleaving enzyme1 (BACE1). On the other hand, flavanones and chalcones isolated from this ginger exhibited higher level of inhibition against the formation of methylglyoxal-derived advanced glycation end-product than the anti-glycating agent, aminoguanidine, indicating that these flavonoids could be beneficial in the prevention and treatment of diabetes (Potipiranun et al., 2018).

Bioactive flavonoids are often of interest in drug discovery. However, the low natural abundance and small quantities of these lead compounds limit the application and drug development in pharmaceuticals. Hence, engineering of flavonoid biosynthetic pathways using transgenic approach and biotransformation techniques were employed to enhance the production of these valuable flavonoids (Li et al., 2015; Munakata et al., 2014; Sasaki et al., 2008; Shen et al., 2012; Zhong et al., 2018). Enhancement of flavonoids can be achieved either by over-expressing regulatory enzymes to up-regulate the pathway, leading to the target compounds and/or by silencing key enzymes to down-regulate the competing pathways. For example, overexpression of a key enzyme in the flavonoid biosynthetic pathway, flavonoid 3′-hydroxylase (F3′H) isolated from *Ginkgo biloba*, was found to enhance the flavonoid production (epigallocatechin, gallocatechin and cathechin) in transgenic poplar plants (Wu et al., 2020). Yin et al. (2020) reported that the production of several flavonoids (liquiritigenin, isoliquiritigenin and isoliquiritin) was enhanced by overexpressing chalcone synthase (CHS) in *Glycyrrhizia uralensis*. Silencing the expression of genes operating in competitive pathway could increase the metabolic flux toward the biosynthesis of desired bioactive metabolites. For example, the amounts of carotenoids present in the petals of Japanese morning glory are very low (Watanabe et al., 2017). When downregulating the key enzyme 9-cis-epoxycarotenoid dioxygenase (NCED) of the abscisic acid biosynthetic pathway using RNA silencing method, Guo et al. (2016) found that the upstream metabolites, mainly the β-carotene and lycopene, have been significantly increased.

Enzymes are typically remarkable specific catalysts. However, a number of enzymes have been reported to be capable of catalyzing other reactions and/or transforming other substrates in addition to the native reaction and substrates (Baier & Tokuriki, 2014; Chen et al., 2015; Cuetos et al., 2016). These unexpected enzymatic properties probably resulted from enzyme promiscuity. In this study, we aimed to enhance the prenylated flavonoid production in *B. rotunda* by over-expressing a prenyltransferase (PTase) gene. A full-length cDNA of PTase from *B. rotunda* (*BrPT2*) was isolated, cloned in a plant expression vector and introduced into *B. rotunda* cell suspension cultures via *Agrobacterium*-mediated transformation. Substrates were biotransformed by the *BrPT2*-expressing *B. rotunda* cells and the product compounds accumulated were analyzed by liquid chromatography-mass spectrometry (LCMS). Unlike any of the previously characterized PTase, BrPT2 showed unprecedented enzyme catalytic promiscuity which resulted in an enhanced yield of other flavonoids.

## Materials and Methods

### Plant material

The cell suspension culture of *B. rotunda* was established according to Wong et al. (2013). Briefly, calli emerged from shoot buds (3–5 cm in height) of *B. rotunda* rhizomes were transferred to propagation media containing Murashige and Skoog (MS) (Murashige & Skoog, 1962) salts supplemented with 3 mg/mL 2,4-dichlorophenoxyacetic acid (2,4-D) and 3% (w/v) mg/L sucrose and 0.2% (w/v) Gelrite (Duchefa Biochemie, Netherlands). After 3 months of culture, the developed calli were transferred to liquid MS medium supplemented with 1 mg/L benzylaminopurine (BAP), 1 mg/L α-napthaleneacetic acid (NAA), 1 mg/L biotin, 2 mg/L 2,4-D, 100 mg/L L-glutamine, and 3% (w/v) sucrose to establish cell suspension cultures. The pH medium was adjusted to pH 5.7. The cell suspension cultures were cultured at 25 °C, 80 rpm under a 16 h light and 8 h dark photoperiod in a growth room. To maintain cell suspension culture, fresh liquid MS liquid medium supplemented with 1 mg/L 2,4-D, 0.5 mg/L BAP and 2% (w/v) sucrose was replaced at a ratio of 1:4 (old to fresh media) at 2 week-intervals.

### Isolation and rapid amplification of cDNA ends (RACE) of *BrPT2*

Total RNA was extracted from *B. rotunda* cells using RNeasy Plant Mini Kit (Qiagen, Hilden, Germany). The quality and concentration of RNA were measured by spectrophotometer (Eppendoff, Enfield, CT, USA). For the 5′ RACE-PCR of *BrPT2*, first strand cDNA was synthesized from the isolated total RNA using the CDS primer A and SMARTScribe Reverse Transcriptase provided by the SMARTer RACE 5′/3′ Kit (Clontech, Tokyo, Japan) according to the manufacturer’s instructions. To amplify the 5′ of the cDNA fragments, gene-specific primers (GSPs), PT2-5′GSP2 (5′ GATTACGCCAAGCTTGGCTTGTTCACTTTGTCAATACCGATGTC 3′) and PT2-5′GSP4 (5′ GATTACGCCAAGCTTCCCAAACAATAAAGACAAGTAATGAATGGACC 3′) were designed against the partial sequence and used with the universal primers (UPM and UPMs). Two rounds of RACE-PCR were performed and the primers for each round were as follows: first round PT2-5′GSP4 and UPM; second round PT2-5′GSP2 and UPMs. The first touchdown PCR was performed using the cycling conditions as follows: 94 °C (30 s) and 72 °C (3 min) for 5 cycles; 94 °C (30 s), 70 °C (30 s) and 72 °C (3 min) for 5 cycles; 94 °C (30 s), 68 °C (30 s) and 72 °C (3 min) for 25 cycles. After the first PCR, the PCR product was diluted 50-fold and re-amplified in a nested PCR reaction under the following condition: 94 °C (30 s), 65 °C (30 s) and 72 °C (1 min) for 20 cycles. PCR product from the nested PCR was purified with NucleoSpin Gel and PCR Clean-Up Kit (Clontech, Tokyo, Japan). The purified PCR products were cloned into pRACE vector (Clontech, Tokyo, Japan) and sequenced.

For the 3′ RACE-PCR of *BrPT2*, first strand cDNA was synthesized from the total RNA of *B. rotunda* using oligo primer and reverse transcriptase SuperScript^®^III (Invitrogen, Carlsbad, CA, USA) according to the manufacturer’s instructions. To amplify the 3′ of the cDNA fragments, two rounds of RACE-PCR were performed using GSPs, PT2-3′GSP (5′ CGAGCTGCATTGGGCCTAACTTTCA 3′) and PT2-3′GSP(N) (5′ TCAGATTTCAACCTTGGCAACAAAG 3′) and universal amplification primer (UAP). Two rounds of RACE-PCR were performed and the primers for each round were as follows: first round PT2-3′GSP and UAP; second round PT2-3′GSP(N) and UAP. The first PCR was performed using the cycling condition as follow: initial denaturation at 94 °C (2 min); followed by 30 cycles of denaturation at 94 °C (20 s), annealing at 56 °C (10 s), and extension at 72 °C (20 s); and a final extension at 72 °C (2 min). About one-fiftieth of the first PCR product was used as template to perform a nested PCR under the same conditions, with a reduced annealing temperature to 55 °C. PCR products from the nested PCR were purified with QIAquick Gel Extraction Kit (Qiagen, Hilden, Germany). The purified PCR products were cloned into pGEM-T Easy vector (Promega, Madison, WI, USA) and sequenced.

### Sequence and phylogenetic analysis

The full-length cDNA sequence and deduced amino acid sequence of *BrPT2* were analyzed using the National Center for Biotechnology Information BLAST programs (http://www.ncbi.nlm.nih.gov/BLAST/). The functional sites or domains in the amino acid sequences were predicted by PredictProtein Server (https://www.predictprotein.org/), iPSORT (http://ipsort.hgc.jp/), ChloroP 1.1 (http://www.cbs.dtu.dk/services/ChloroP/) and TargetP1.1. The molecular weight (MW) and theoretical isoelectric point (pI) were predicted using Compute pI/MW (https://web.expasy.org/compute_pi/). Multiple sequence alignments and phylogenetic-tree construction applying the neighbor-joining method were conducted using the ClustalW program (http://www.ebi.ac.uk/clustalW) and MEGA 7.0 software, respectively.

### Construction of plant expression plasmid

In-Fusion cloning (Takara, Shiga, Japan) was employed to clone *BrPT2* gene into the modified plant expression plasmid pRI909 (Takara, Shiga, Japan). PCR of insert *BrPT2* was performed using primers IFPT2FW (5′ CCATCACCATCACCATCACCTCGAATCAATGGCTCCTTCTCACCAAGC 3′) and IFPT2RV (5′ CCAAATATTTCATCTTCATCTTCATATCTATATGAAAGGAAAAATAATG 3′) with Primstar HS DNA polymerase (Takara, Shiga, Japan) and PCR cycling conditions were as follow: initial denaturation at 98 °C (5 s); followed by 25 cycles of denaturation at 98 °C (10 s), annealing at 62 °C (5 s), and extension at 72 °C (1 min 30 s). To amplify the vector, KOD FX Neo DNA polymerase (Toyobo, Osaka, Japan) and primer 5UTRRV (5′ TGATTCGAGGTGATGGTGATGGTGATGGTAGTACGACATCTTTAATCTTGATTTGATTAAAAGTTTATAT 3′) were used. PCR cycling conditions were as follow: initial denaturation at 94 °C (2 min); followed by 30 cycles of denaturation at 98 °C (10 s), annealing at 60 °C (30 s), and extension at 68 °C (6 min). A poly-histidine tag was fuzed at the N-terminal of the *BrPT2*. After verifying the vector and insert, in-fusion cloning was carried out using in-fusion cloning kit (Takara, Shiga, Japan) according to manufacturer’s instruction. The reaction mixture was then transformed into *E. coli* DH5α. Positive insert that has been confirmed by colony PCR was selected for plasmid extraction by using QIAprep Spin Miniprep kit (Qiagen, Hilden, Germany). The plasmid was sent for sequencing.

### Transformation of the binary vector into *Agrobacterium tumefaciens*

About 1 µg of *pRI-BrPT2* plasmid DNA was added into 100 µL of *Agrobacterium tumefaciens* strain LBA4404 competent cell culture and kept on ice for 30 min before frozen in liquid nitrogen. The mixture was immediately heat-shocked at 37 °C for 4 min and incubated on ice for 1 min. Next, 1 mL of Luria-Bertani medium was added into the mixture and incubated at 28 °C for 2 h. The mixture was then centrifuged at 12,000 rpm for 2 min before the pellet was suspended in 50 µL LB medium. The mixture was plated on LB medium containing 100 µg/mL streptomycin and 100 µg/mL kanamycin and incubated at 28 °C for at least 48 h.

### Plant transformation

*Agrobacterium tumefaciens* strain LBA4404 harboring *pRI-BrPT2* was grown on LB agar supplemented with 100 mg/L kanamycin and 100 mg/L streptomycin and incubated at 28 °C for 2 days in dark. A single bacterial colony was selected and inoculated in 10 mL LB broth supplemented with the same antibiotics and incubated at 28 °C, gently agitated at 120 rpm for 16 h in dark. About 1 mL of overnight bacterial culture was inoculated into 20 mL LB supplemented with the same antibiotics and incubated at 28 °C with shaking at 120 rpm in dark until its optical density at 600 nm (OD600) reached ~1.0. The 14 day-old *B. rotunda* settled cells (1 mL) were infected with *Agrobacterium* culture supplemented with 100 µm acetosyringone for 10 min at 28 °C under shaking condition of 120 rpm. The *Agrobacterium*-containing medium was replaced with 10 mL of fresh liquid MS medium supplemented with 100 µm acetosyringone. The infected cells were co-cultivated with *Agrobacterium* culture at 28 °C in dark for 3 days. The co-cultivation medium was replaced with fresh liquid MS medium containing 300 mg/L cefotaxime to eliminate excessive *Agrobacterium*. The cells were selected on 50 mL liquid MS medium containing the 300 mg/L cefotaxime and 50 mg/L kanamycin for 28 days with replacement of new liquid MS medium containing the 300 mg/L cefotaxime and 50 mg/L kanamycin every 7 days before PCR amplification and biotransformation.

### DNA extraction and PCR amplification

Genomic DNA of the transformed *B. rotunda* cells with pRI-*PT2* transgene was extracted using DNeasy Plant Mini kit according to the manufacturer’s protocol (Qiagen, Hilden, Germany). PCR was performed to amplify the pRI-*PT2* transgene with an expected size of 1,200 bp. Primers 5′UTRFW (5′ TAATCAAATCAAGATTAAAGCCATCACCATCACCATCACCTCGAATCAATGGCTCCTTCTCACCAAGC 3′) and TERRV (5′ CCAAATATTTCATCTTCATCTTCATATCTATATGAAAGGAAAAATAATG 3′) were designed from the 5′ UTR region of the vector and the gene terminator region. The reaction conditions were as follows: an initial denaturation at 95 °C for 5 min; 35 cycles of denaturation at 94 °C for 30 s, annealing at 55 °C for 30 s and extension at 72 °C for 1 min; and a final elongation step at 72 °C for 10 min.

### Protein extraction and Western blot analysis

The transgenic and wild type (control) cells were harvested and ground into fine powder in the presence of liquid nitrogen. The fine powder was dissolved in extraction buffer (50 mM HEPES-KOH, pH 7.5, 50 mM NaCl, 0.5% Triton X-100, 10% glycerol, 1 × protease inhibitor cocktail, and 1% PVP) at a ratio of 100 mg:100 µL. The mixture was incubated at 4 °C for 30 min followed by centrifugation at 13,000×*g* for 5 min at 4 °C. The supernatant was transferred into a new 1.5 mL microcentrifuge and the crude extract fraction was subjected to the affinity chromatography using nickel-chelating resin, Ni-NTA superflow (Qiagen, Hilden, Germany). The purified His-tag fusion protein was concentrated and exchanged with storage buffer (10 mM Tris-HCl, pH 7.5, 10 mM MgCl_2_) using an Amicon Ultracel Column (Millipore, Billerica, MA, USA). Protein content was measured using Bradford assay (Bradford, 1976). The purified protein of 1 µg was separated by 12% SDS–PAGE and then transferred to a polyvinylidene difluoride membrane (GE Healthcare, Chicago, IL, USA). The presence of poly-histidine-tag BrPT2 protein was verified by HisDetector™ Western Blot AP Colorimetric kit (KPL).

### Biotransformation by *B. rotunda* cell suspension cultures

Two substrates, namely 100 μM pinostrobin chalcone and 100 μM geranyl diphosphate (GPP), were added to wild-type and transgenic *B. rotunda* cell suspension cultures and incubated at room temperature for 3 days with gentle rocking. The cells were harvested by filtering and immediately frozen in liquid nitrogen before stored at −80 °C.

### Isolation of flavonoids and LC/MS analysis

Biotransformed *B. rotunda* cells were freeze-dried for 2 days and then (500 mg) extracted using 5 mL methanol. The mixture was sonicated for 5 min at 37 kHz before incubated at 4 °C overnight. The extraction process was repeated twice. The methanol extracts were evaporated and partitioned with an equal volume of ethyl acetate (EA) and water. The EA fractions were evaporated to dryness and the crude extracts were dissolved in methanol. The samples were subsequently filtered through a 0.45 μm PTFE filter. The methanol extracts from transgenic and wild-type cells were analyzed by using Shimadzu Prominence UFLC system coupled with AB SCIEX QTRAP 5500 mass spectra detector. Mass spectra were acquired in positive electrospray ionization modes. The separation was carried out using Kinetex 5 µm EVO C18 100 Å LC column (150 × 2.1 mm) with corresponding solvent A (0.1% formic acid (FA) in water) and solvent B (0.1% FA in acetonitrile). Compounds were eluted using a linear gradient from 5% to 95% solvent B for 8 min at a flow rate of 0.2 mL/min Mass spectra were acquired in multiple reaction monitoring with positive electrospray ionization mode. The concentration of analytes was determined by interpolation of the relative peak areas for each analyte to internal standard peak area in the sample on the spiked calibration curve. In order to compensate for losses during sample processing and instrumental analysis, an internal standard was used. The content of flavonoids in samples were quantified in µg/ml. Six representative compounds of the phenylpropanoid pathway: naringenin (retention time = 4.67, m/z, 273.2, 153.1, 131.1), cardamonin (retention time = 5.44, m/z 271.1, 167.2, 124.2), alpinetin (retention time = 4.9, m/z 271.1, 167.1, 131.2), panduratin A (retention time = 6.09, m/z 407.1, 167.0, 83), pinostrobin (retention time = 5.58, m/z 271.2, 167.0, 131.0) and pinocembrin (retention time = 5.17, m/z 257.1, 153.1, 131.1) were used as standard. Analyst software version 1.6.3 was used to process the data.

### In-vitro screening of potential substrates

Various substrates (pinostrobin, pinocembrin, naringenin, cardamonin, pinostrobin chalcone, 2′-hydroxychalcone, 2′-Hydroxy-4-bromo-4′,6′-dimethoxychalcone, 2′-Hydroxy-4′-methoxychalcone, 2-Cyclohexenol, 2-Cyclopentenol) with different co-factors (dimethylallyl pyrophosphate; DMAPP and geranly pyrophosphate; GPP) were used for substrate specificity analysis of recombinant BrPT2. The reaction contained 1 µg of BrPT2 purified protein, 10 mM Tris-HCl (pH7.5), 10 mM MgCl_2_, 1 × protease inhibitor cocktail and different substrates at concentration of 200 µM. The reaction mixtures were incubated in a total volume of 250 µL at 25 °C for 16 h. The enzymatic reaction mixtures were analyzed by Agilent G6550A LC/Q-TOF MS coupled with electrospray ionization (ESI–MS). Mass spectra were acquired in negative and positive electrospray ionization modes. Separation was carried out using ZORBAX Eclipse Plus C18 3.5 µm (100 × 4.6 mm) with corresponding solvent A (0.1% FA in acetonitrile) and solvent B (0.1% FA in water). Compounds were eluted using an isocratic profile of 100% solvent A for 10 min at a flow rate of 0.2 mL/min.

### Enzymatic assay and reaction product quantification

The enzymatic assay reaction contained 10 mM Tris-HCl (pH 7.5), 10 mM MgCl_2_, 200 µM pinostrobin chalcone, 200 µM GPP or 200 µM DMAPP and BrPT2 protein (Table 1). Negative control reaction containing all components but exclude BrPT2 protein was also set-up. The reaction mixtures were incubated in a total volume of 250 µL at 25 °C for 16 h. The products were quantified using Shimadzu Prominence UFLC system coupled with AB SCIEX QTRAP 5500 mass spectra detector. Mass spectra were acquired by multiple reaction monitoring in negative and positive electrospray ionization modes. The concentration of analytes was determined by interpolation of the relative peak areas for each analyte to internal standard peak area in the sample on the spiked calibration curve. In order to compensate for losses during sample processing and instrumental analysis, an internal standard was used. The enzymatic product were quantified in µg/ml. Mass spectra were acquired in negative and positive electrospray ionization modes. The separation was carried out using Kinetex 5 µm EVO C18 100 Å LC column (150 × 2.1 mm) with corresponding solvent A (0.1% FA in water) and solvent B (0.1% FA in acetonitrile). Compounds were eluted using a linear gradient from 5% to 95% solvent B for 8 min at a flow rate of 0.2 mL/min.

**Table 1:** In-vitro enzyme assay reactions.

Components	S1	S2	S3	S4	S5	S6
10 mM TRIS-HCl	/	/	/	/	/	/
10 mM MgCl2	/	/	/	/	/	/
100X Protease inhibitor cocktail	/	/	/	/	/	/
200 μM Pinostrobin chalcone	/	/	/	/	/	/
200 μM GPP	/	X	X	/	/	X
200 μM DMAPP	/	X	X	/	X	/
BrPT2 purified protein	X	/	X	/	/	/

**Notes:**

/ with.

X without.

### Statistical analysis

All values were presented as mean ± standard deviation of three independent experiments. The differences in compound accumulation in wild type and *BrPT2*-expressing transgenic cells (T1) were compared using Student’s *t*-test. A *p* value of less than 0.05 was considered statistically significant. Data analysis of the enzymatic reaction product of recombinant BrPT2 protein was done using one-way ANOVA and Tukey’s test. All analyses were conducted using SPSS software (IBM Corp. Released 2017. IBM SPSS Statistics for Windows, Version 25.0. Armonk, NY, USA: IBM Corp.).

## Results

### Molecular cloning and sequence analysis of *BrPT2*

**Figure 1:**
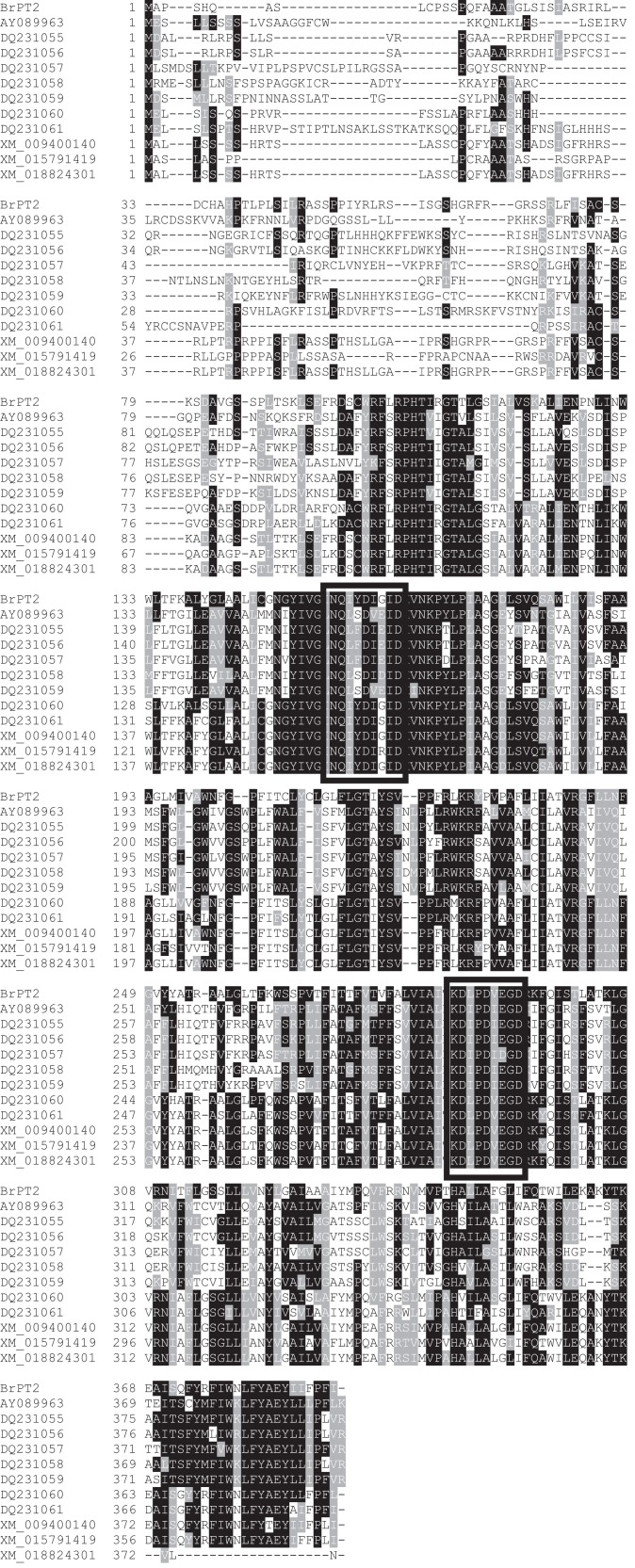
Comparison of the amino acid sequence of *Boesenbergia rotunda*, BrPT2 with other prenyltransferases.

In this study, we obtained full-length cDNA of *B. rotunda PT2* gene (designated as *BrPT2*) from a partial unigene gene sequence (Md-Mustafa, 2017) using 5′ and 3′ RACE. The ORF of *BrPT2* cDNA was 1,176 bp, encoding a single peptide of 392 amino acid residues with a predicted MW of 43.4 and the predicted pI was 9.92 (GenBank accession no. MN564943; Fig. S1). The deduced amino acid sequence of BrPT2 shared high identities (81%) with the predicted homogentisate phytyltransferase 2 chloroplastic isoform X1 from *Musa acuminata* subsp. *malaccensis* (accession number: XM_009400140). Two conserved characteristic motifs (NDXXDXXXD and NQXXDXXXD) which are similar to plant-derived prenyltransferase were found (Fig. 1). When we aligned the amino acid sequence of BrPT2 with the recently published chalcone isomerase, BrCHI (GenBank accession number: MK421357), we found that only less than 20% similarity between these two amino acid sequences (Fig. S2; Chia, Teh & Mohamed, 2020). BrPT2 does not contain the conserved active side of chalcone isomerase protein family as reported in previous studies (Cheng et al., 2011; Shimada et al., 2003; Wei et al., 2019; Chia, Teh & Mohamed, 2020) but this protein has 9 transmembrane α-helices and possesses a putative chloroplast transit peptide at its N-terminal.

**Figure 2:**
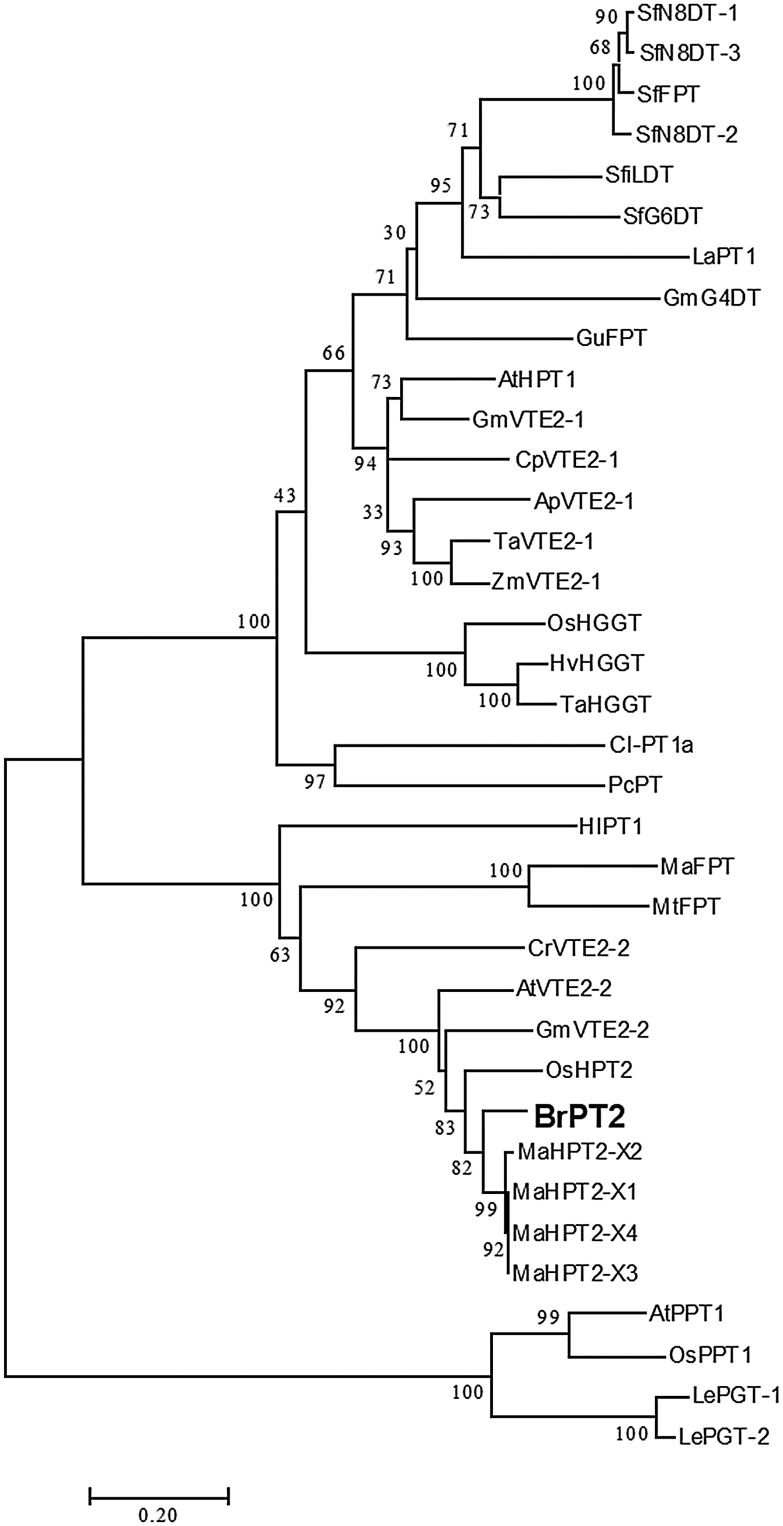
Phylogenetic tree of BrPT2.

A neighbor-joining phylogenetic tree was constructed using deduced amino acid sequences of BrPT2 to analyze the relationship of BrPT2 with other plant-derived PTs (Fig. 2). The deduced amino acid sequence of BrPT2 indicated that it shared identity with PTs involved in plastoquinone biosynthesis (XM015791419; DQ231061; DQ231060; AM285678) but does not belong to any of the plant PT protein clusters.

### Overexpression of *BrPT2* in *B. rotunda* cells

The full-length cDNA of *BrPT2* isolated from *B. rotunda* was cloned into pRI vector and expressed in *B. rotunda* cell suspension cultures via *Agrobacterium*-mediated transformation. Genomic DNA from the putative transformants was extracted and analyzed by PCR amplification using gene-specific primers to verify the positive transformants. A single band at ~1.2 KB was detected based on gel electrophoresis, confirming the presence of the *BrPT2* in the transformed cells (Fig. S3).

### LC/MS analysis of flavonoids in transgenic *B. rotunda* cells

**Figure 3:**
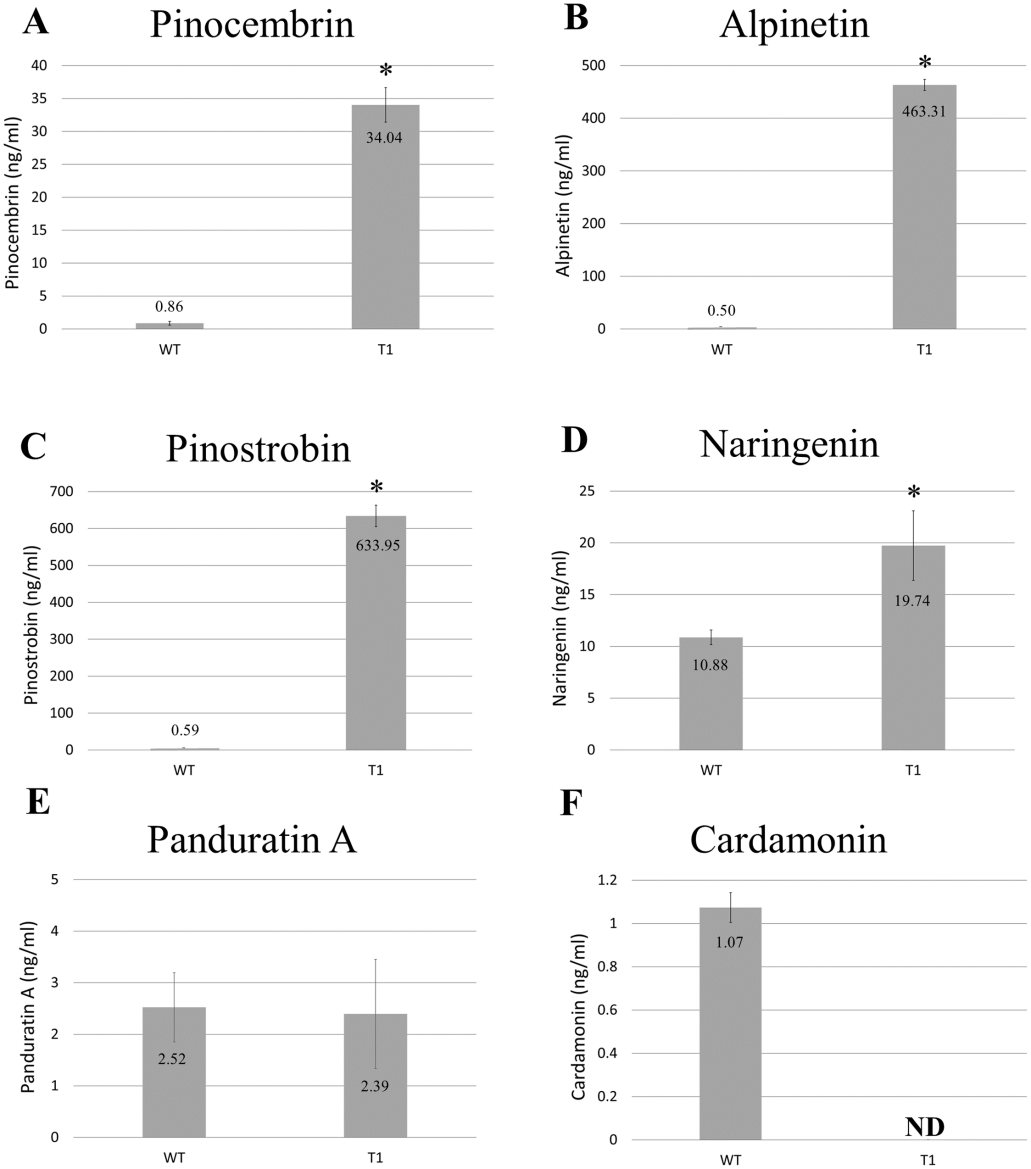
Metabolite analysis of *B. rotunda* cells transformed with *BrPT2*. Levels of compounds in wild type and transgenic (T1) expressing *BrPT2*. (A) Pinocembrin, (B) Alpinetin, (C) Pinostrobin, (D) Naringenin, (E) Panduratin, (A and F) Cardamonin. Statistical analysis was performed using Student’s *t* test, and a significant difference compared to wild type is indicated by an asterisk (*p* < 0.05). N.D: not detected. Error bars represent standard deviations for three biological replicates.

Methanolic extracts from freeze-dried wild-type and transgenic *B. rotunda* cells were quantitatively analyzed using LC/MS for six representative flavonoids of the phenylpropanoid pathway: naringenin, cardamonin, alpinetin, panduratin A, pinostrobin and pinocembrin, as standards. Our results showed the content of alpinetin (463.31 ng/ml), pinostrobin (633.95 ng/ml), pinocembrin (34.04 ng/ml) and naringenin (19.74 ng/ml) in *BrPT2*-expressing transgenic cells were significantly higher (*p* < 0.05) than those observed in the wild type cells (alpinetin, 0.50 ng/ml; pinostrobin, 0.59 ng/ml; pinocembrin, 0.86 ng/ml and naringenin, 10.88 ng/ml, respectively) (Fig. 3). However, panduratin A content remained unchanged and cardamonin was not detected in transgenic *B. rotunda* cells.

### Purification and immunoblotting of BrPT2 from transgenic *B. rotunda* cells

**Figure 4:**
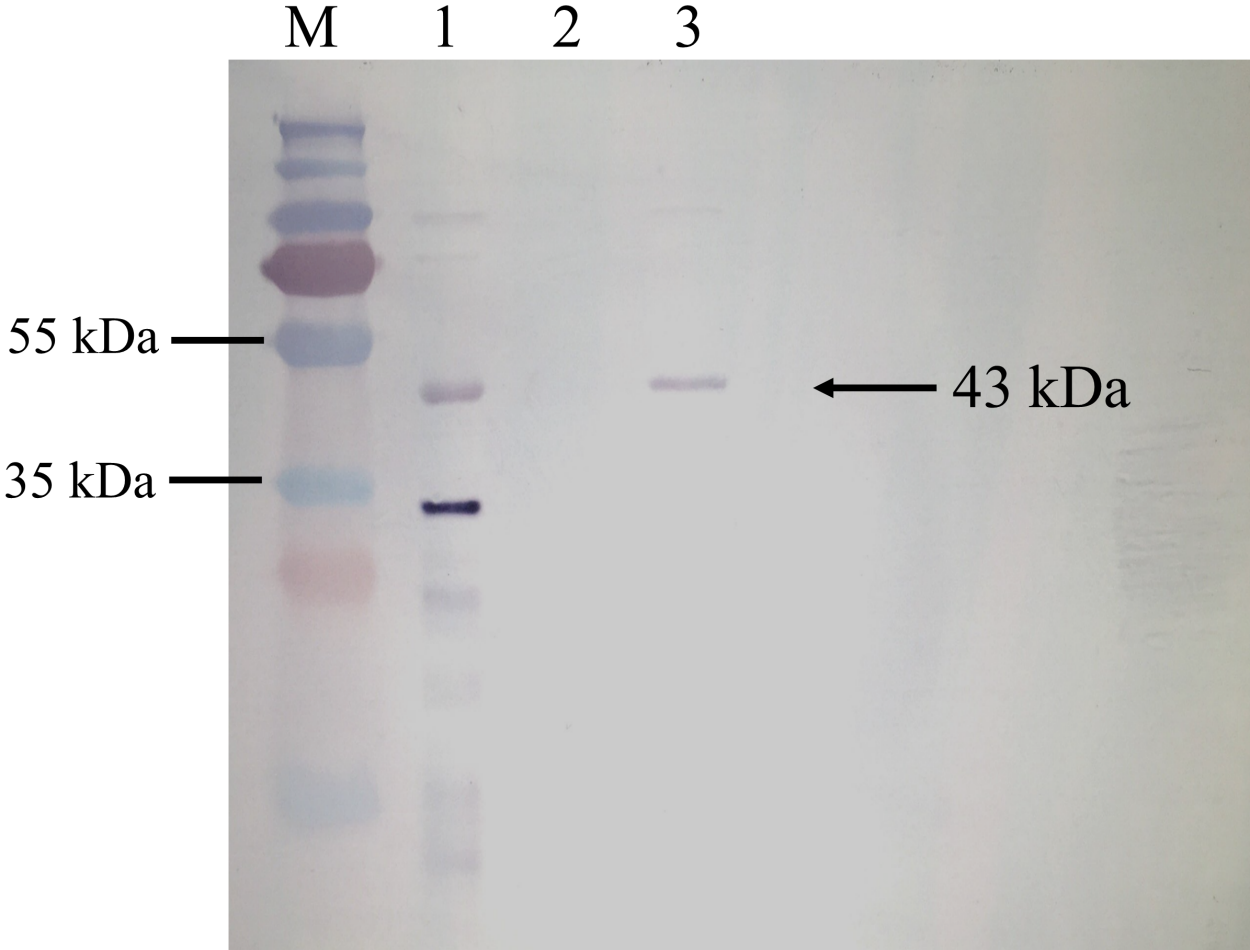
Western blot analysis of BrPT2 recombinant protein expressed in *B. rotunda* cell suspension culture. The purified protein was separated on 12% SDS–PAGE, transferred to PVDF membrane and incubated with HisDetector Nickel-AP conjugate. M: protein marker; Lane 1: total protein of BrPT2; Lane 2: wild type (negative control); Lane 3: purified BrPT2 protein. Arrow indicates the target protein with molecular weight of ~43 kDa.

BrPT2 from transgenic *B. rotunda* cells was isolated, purified and detected by immunoblotting with His-antibody. As shown in Fig. 4, a single band with a MW of ~43 kDa was observed in the total and purified protein fraction, whereas no signal was observed in the wild type (negative control), indicating the target protein was successfully expressed. The purified BrPT2 protein was then used for in-vitro enzyme assay.

### Identification of enzyme activity with substrates

**Figure 5:**
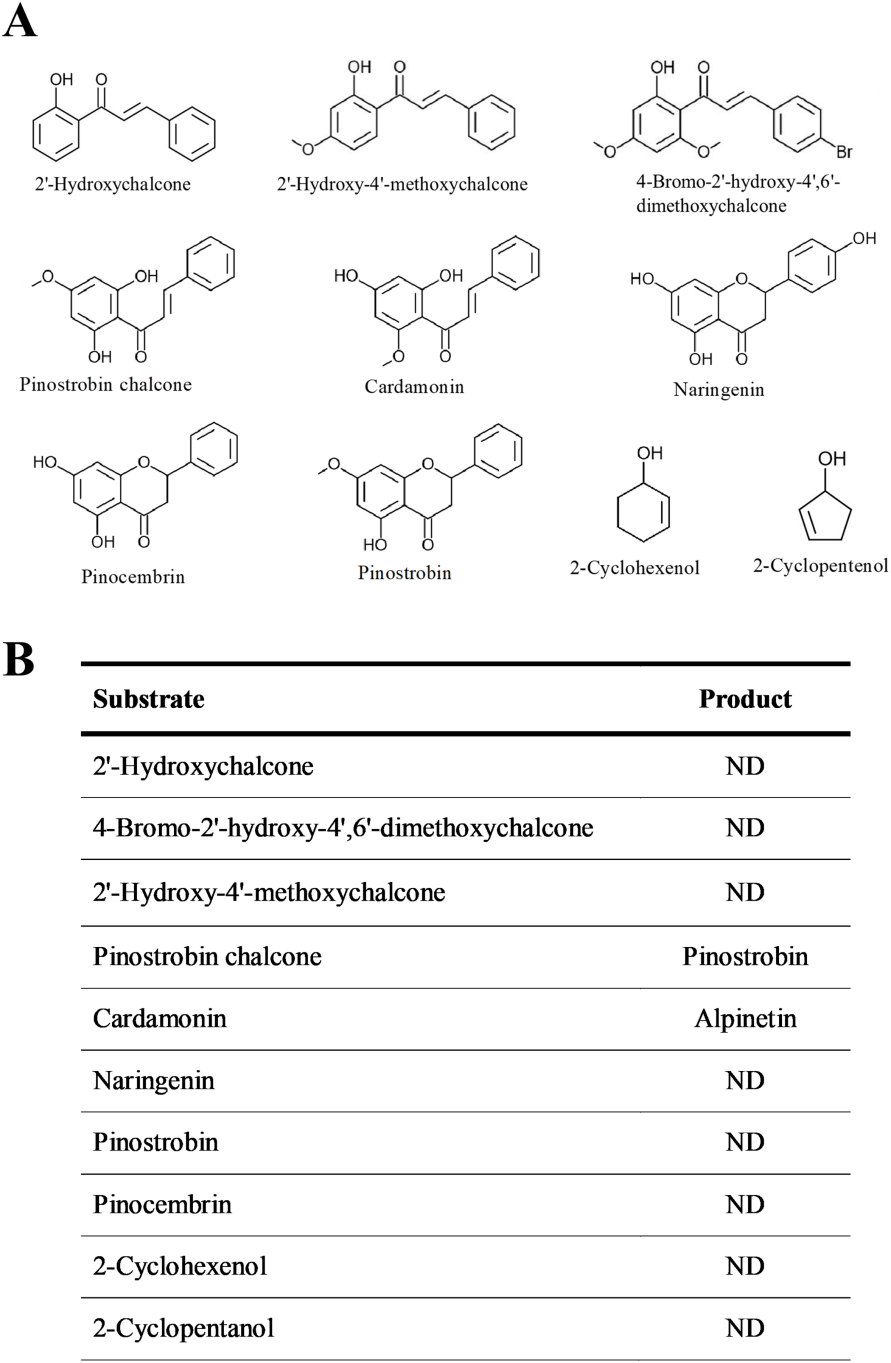
Substrate specificity of recombinant BrPT2. (A) Chemical structures of substrates used in the experiments. (B) Substrates and reaction products. ND: not detected.

The substrate specificity of BrPT2 was analyzed using ten substrates at the same concentration (Fig. 5). The reaction products of the enzyme assays were identified by directly comparing it with standards. Of the substrates tested, only pinostrobin chalcone and cardamonin were catalyzed by recombinant BrPT2 to form pinostrobin and alpinetin, respectively.

**Figure 6:**
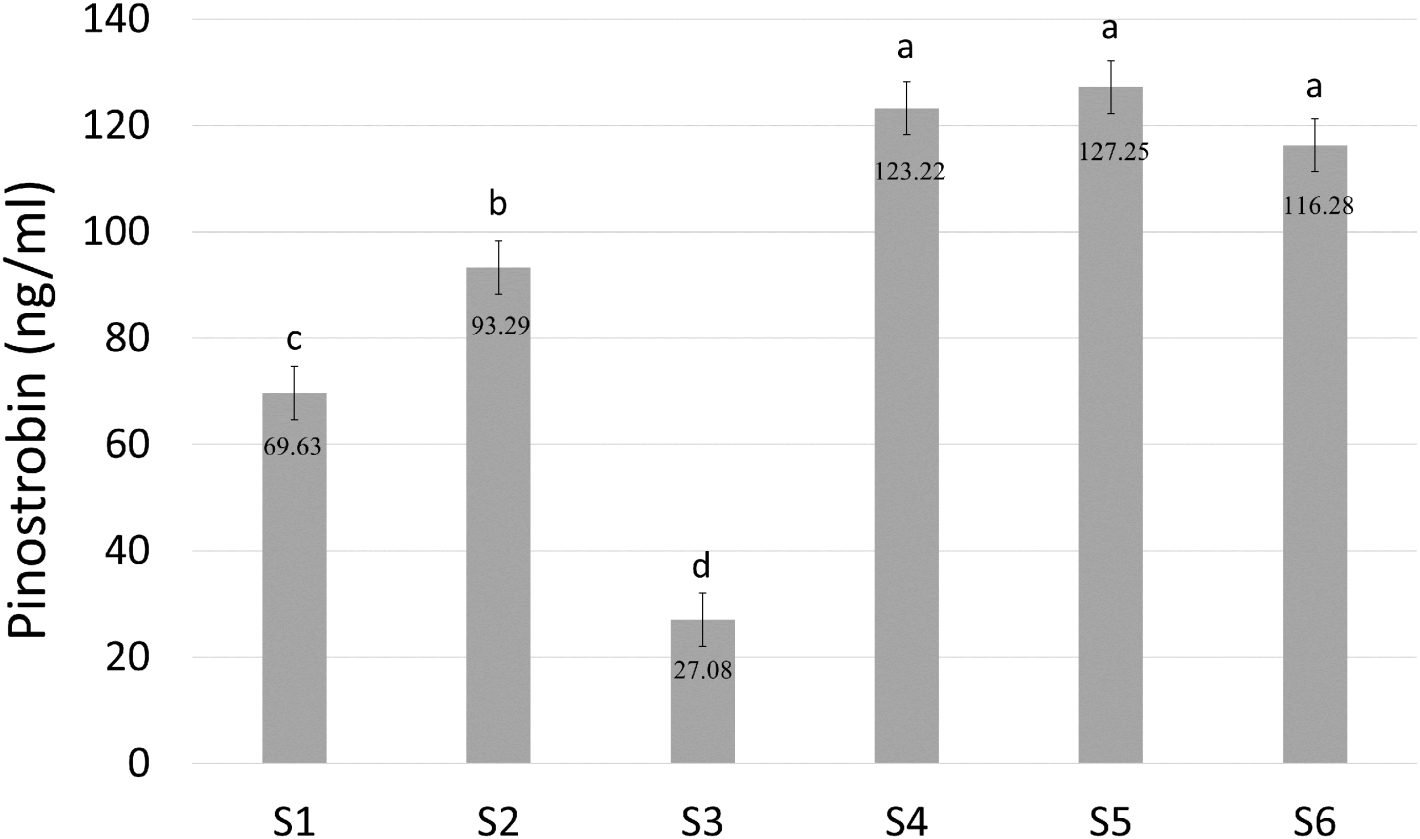
Quantification of amount of pinostrobin in an enzymatic assay containing with or without recombinant BrPT2 protein isolated from transgenic *B. rotunda* cells and GPP or DMAPP. Pinostrobin ion[M+H]^+^ at m/z 271.2, 167.0. S1–S6 refer to different combination assays incubated either with or without BrPT2 and negative controls as indicated in Table S1. Values are means ± SD for triplicate experiment. Different letters indicate significant differences at 95% by Tukey’s multiple comparison test.

Pinostrobin chalcone ([M−H]^−^ at m/z 269.2, 165.2) was selected for further analysis to determine the enzymatic activity of BrPT2 in catalyzing pinostrobin chalcone to pinostrobin ([M+H]^+^ at m/z 271.2, 167.0) in in-vitro assay. As shown in Fig. 6, S4, S5 and S6 produced the highest accumulation of pinostrobin at 123.22 ng/ml, 127.25 ng/ml and 116.28 ng/ml, respectively, followed by S2 (93.29 ng/ml). In contrast, the reaction without BrPT2 (S1 and S3) produced a lower amount of pinostrobin compared to those contained BrPT2. The amount of pinostrobin was 69.63 ng/ml in S1 (without BrPT2) and 27.08 ng/ml in S3 (without BrPT2 protein, GPP and DMAPP). This suggested that BrPT2 could accelerate the enzymatic reaction, leading to the accumulation of a higher amount of pinostrobin compared to reaction without BrPT2, at the harvesting time. Besides, GPP and DMAPP were tested as prenyl donor and pinostrobin chalcone as prenyl acceptor, and no significant difference in the amount of pinostrobin produced was detected. This suggests that BrPT2 does not exhibit specific specificity towards prenyl donors, such as GPP and DMAPP, in order for the addition of prenyl group to chalcones.

## Discussion

Prenylated flavonoids via prenylation process, which is catalyzed by PTase, exhibited a wide variety of bioactivities (Busch et al., 2015; Zelová et al., 2014). However, only a few flavonoid-related *PT* genes have been identified so far. We isolated full length cDNA of *BrPT2* from *B. rotunda* and analyzed its relationship with PTase from other plant species. We found that the isolated BrPT2 shared the highest similarity of amino acid sequences with *Musa acuminata* homogentisate phytyltransferase and was placed in the same clade with phytyltransferases from other plant species. However, BrPT2 formed a distinctive branch from all other phytyltransferases, suggesting that BrPT2 may have evolved independently in the plant lineages.

PTase are involved in transferring prenyl moiety to a wide range of prenyl acceptors, such as other prenyl moiety, proteins, phenolic or aromatic compounds (Brandt et al., 2009). Aromatic PTases (transferring prenyl moiety to aromatic compounds) play a major role in the diversification of most aromatic secondary metabolites and are divided into soluble and membrane-bound PT (Yazaki, Sasaki & Tsurumaru, 2009). In this study, we found that the isolated *BrPT2* consists of an aspartate rich motif (NDXXDXXXD and NQXXDXXXD) which is one of the important membrane-bound PT characteristics.

We then investigated the role of *BrPT2* in the flavonoid biosynthetic pathway (Fig. S4) by expressing this gene in *B. rotunda* cell suspension cultures and analyzed its function through in-vivo biotransformation assay. We fed the transgenic *BrPT2*-expressing *B. rotunda* cells with pinostrobin chalcone and harvested the cells for LC/MS analysis. Prenylated products for the selected flavonoids were not detected in the analysis probably due to the substrate specificity or unfavorable substrate. While we did not detect any prenylated flavonoids, we observed that other flavonoid products, namely alpinetin, naringenin, pinostrobin and pinocembrin, were significantly higher in transgenic *BrPT2*-expressing *B. rotunda* cells than the wild type. The results seem to indicate that isomerization and cyclization reactions to be more favorable for pinostrobin chalcone to be converted to the aforementioned flavonoids. Although cardamonin was not detected in *BrPT2*-expressing *B. rotunda* cells, we observed that the amount of cardamonin end-product, alpinetin, was higher than that in the wild type. Cardamonin, a metabolic intermediate associated with the phenylpropanoid pathway, was most likely being converted to form alpinetin. To confirm the substrate specificity and enzymatic reaction of BrPT2, different combinations of substrates were tested with BrPT2 protein in in-vitro assay according to previous published studies (Karamat et al., 2014; Li et al., 2015; Shen et al., 2012). The results of the substrate specificity assay showed that among all substrates tested, BrPT2 were able to convert pinostrobin chalcone and cardamonin into pinostrobin and alpinetin, respectively (Fig. 5). Further analysis using other chalcones (2′-hydroxychalcone, 2′-hydroxy-4-bromo-4′,6′-dimethoxychalcone, 2′-hydroxy-4′-methoxychalcone) and some simple molecules (2-cyclohexenol, 2-cyclopentenol) that are prone to undergo cyclization or isomerization were also tested. However, no activity was detected in the assay. These in-vitro assay findings suggested that BrPT2 catalyzed the synthesis of pinostrobin chalcone into pinostrobin and cardamonin into alpinetin but did not react with other substrates tested in this study.

Interestingly, the in-vitro enzymatic results (Fig. 6) were in agreement with the results obtained from the in-vivo biotransformation assay (Fig. 3). Only two chalcones (pinostrobin chalcone and cardamonin) that are present in the *B. rotunda* phenylpropanoid pathway were catalyzed by BrPT2 to form pinostrobin and alpinetin, respectively, suggesting that the enzymatic reaction and biotransformation process may have led to high product accumulation from the isomerization and cyclization reactions facilitated by BrPT2. This might be due to the substrate specificity of the BrPT2 where the BrPT2 seems to favor specific chalcones derived from the phenylpropanoid pathway in *B. rotunda*. When tested with other chalcones that are not present in *B. rotunda* (2′-hydroxychalcone, 2′-Hydroxy-4-bromo-4′,6′-dimethoxychalcone and 2′-Hydroxy-4′-methoxychalcone), we failed to detect any corresponding products.

The findings of this study suggest that unlike any of the previously characterized PTase, BrPT2 showed unprecedented enzyme catalytic promiscuity in this study which resulted in enhanced flavanones instead of prenylated flavonoids. Enzyme promiscuity refers to the capability of catalyzing alternative reactions in addition to its native catalysis reaction. Enzyme catalytic promiscuity indicates the ability of an enzyme to catalyze distinctly different chemical reactions whereas substrate promiscuity enables the enzyme to show a broad substrate specificity (Copley, 2017; Guzmán et al., 2019; Khersonsky & Tawfik, 2010; Nobeli, Favia & Thornton, 2009). Upon introduction of the heterologous gene into a biological system, promiscuous activities of the heterologous enzymes may cause undesirable byproducts accumulation via alternative catalytic routes. However, promiscuous enzyme activities of heterologous genes are often neglected in the study of metabolic pathways. Some enzymes have been reported to catalyze reaction other than the native reaction (Moghe & Last, 2015; Weng & Noel, 2012). For example, the type IV chalcone isomerase-like protein (CHIL) that lacks chalcone isomerase activity has been reported to enhance the flavonoid production in many plant species. The mechanism of CHIL in catalyzing flavonoid production remains unknown (Ban et al., 2018; Jiang et al., 2015; Morita et al., 2014). We speculate that BrPT2 could be a catalytic promiscuous enzyme as it did not perform its native function which is prenylation but instead it showed isomerization activity. Although BrPT2 might have similar function with chalcone isomerase (Figs. 3 and 6), the protein sequence identity with the recently published chalcone isomerase from *B. rotunda* was very low (<20%) (Chia, Teh & Mohamed, 2020). Moreover, the isolated BrPT2 does not contain the conserved amino acid residues that are essential for stereospecific isomerization (Cheng et al., 2011; Chia, Teh & Mohamed, 2020; Shimada et al., 2003; Wei et al., 2019).

To the best of our knowledge, this is the first observation of BrPT2 promiscuity where pinostrobin chalcone and cardamonin was converted to pinostrobin and alpinetin, respectively, instead of prenylated products in metabolically engineered ginger. A similar observation was made by Yun et al. (2018) where heterologous enzymes functionally expressed in *Saccharomyces cerevisiae* showed promiscuous activities that led to unintended metabolic rerouting. Heterologous enzymes, xylose reductase, xylitol dehydrogenase and β-glucosidase demonstrated promiscuous activities by unexpectedly converting galactose, galactitol and cellobiose into galactitol, tagatose and trisaccharide, respectively (Yun et al., 2018). In the case of prenyltransferase, enzyme-substrate promiscuity had been reported in an aromatic prenyltransferase from *Aspergillus terreus*. The mentioned prenyltransferase was observed with substrate promiscuity toward a wide range of aromatic acceptors and prenyl donors, generating products of different prenylation patterns (Chen et al., 2017).

## Conclusions

In summary, the expression of *BrPT2* in *B. rotunda* cell suspension cultures showed significant enhanced accumulation of several flavonoids in the phenylpropanoid biosynthetic pathway. We speculated that BrPT2 may have catalytic enzyme promiscuity based on the unexpected conversion of pinostrobin chalcone into pinostrobin and cardamonin into alpinetin. In addition, biotransformed *B. rotunda* cells produced higher levels of pinostrobin, pinocembrin, alpinetin and naringenin as compared to wild type. Results from the experiments indicated that catalytic enzyme promiscuous activity of BrPT2 favored substrates pinostrobin chalcone and cardamonin. However, the mechanism or mode of action of *BrPT2* in catalyzing pinostrobin chalcone to prenylated flavonoids, still remain unknown. This study, however, has provided useful information for the improvement of *B. rotunda* flavonoid production and systematic optimization of *B. rotunda* metabolic pathway to manufacture therapeutically useful bioactive products.

## Supplemental Information

10.7717/peerj.9094/supp-1 Supplemental Information 1 RACE-PCR and PCR amplification of the full-length *BrPT2* gene. (A) 3′ RACE with ~550 bp target product. (B) 5′ RACE with ~650 bp target product. (C) Full-length PCR product of *BrPT2*. M: molecular weight marker. (A) 3′ RACE with ~550 bp target product. (B) 5′ RACE with ~650 bp target product. (C) Full-length PCR product of *BrPT2*. M: molecular weight marker.

10.7717/peerj.9094/supp-2 Supplemental Information 2 Amino acid sequence alignment of prenyltransferase isolated from *Boesenbergia rotunda*, BrPT2, with chalcone isomerase isolated from *Boesenbergia rotunda*, BrCHI (Accession number: MK421357). Conserved active sides of chalcone isomerase are show. Conserved active sides of chalcone isomerase are shown in white on black background.

10.7717/peerj.9094/supp-3 Supplemental Information 3 Genomic DNA of pRI-*BrPT2 and PCR amplification of transgenic B. rotunda* *cells*. M: DNA marker; Lanes 1 and 2: genomic DNA of transformed cells. Lane M: molecular weight marker, Lanes 1 and 2: single band at ~1.2 KB was detected in positive transformants, Lane 3: wild type, and Lane 4: positive control.

10.7717/peerj.9094/supp-4 Supplemental Information 4 The proposed biosynthetic pathway for flavonoids and chalcones in *B. rotunda*.

10.7717/peerj.9094/supp-5 Supplemental Information 5 Metabolite analysis of *B. rotunda* cells transformed with *BrPT2*. Levels of compounds in wild type and transgenic (T1) expressing *BrPT2*.

10.7717/peerj.9094/supp-6 Supplemental Information 6 Quantification of amount of pinostrobin in an enzymatic assay containing with or without recombinant BrPT2 protein isolated from transgenic *B. rotunda* cells and GPP or DMAPP.

10.7717/peerj.9094/supp-7 Supplemental Information 7 BrPT2 gene sequence.

## Additional Information and Declarations

### DNA Deposition

The following information was supplied regarding the deposition of DNA sequences: The prenyltransferase, PT2 gene (designated as BrPT2) is available at GenBank: MN564943.

### Data Availability

The following information was supplied regarding data availability: The raw measurements are available in the Supplemental Files.
