# Supplementary figures and images for: Enhancing flavonoid production by promiscuous activity of prenyltransferase, BrPT2 from Boesenbergia rotunda
Source: PeerJ. 2020 May 1;8:e9094. doi: 10.7717/peerj.9094 (PMC7197402; doi:10.7717/peerj.9094)

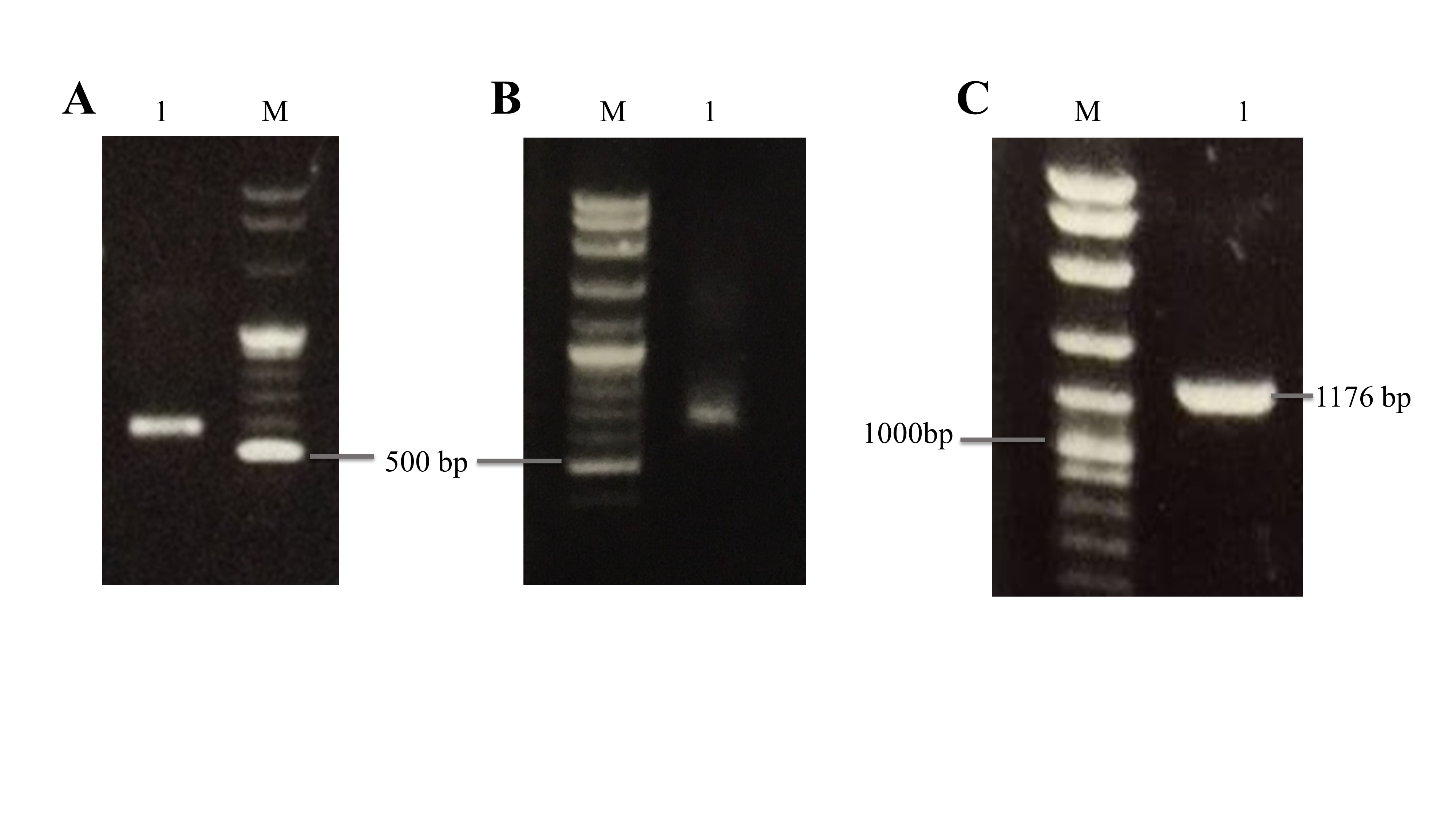

Supplement: Supplemental Information 1 — (A) 3′ RACE with ~550 bp target product. (B) 5′ RACE with ~650 bp target product. (C) Full-length PCR product of BrPT2. M: molecular weight marker. [file peerj-08-9094-s001.png]

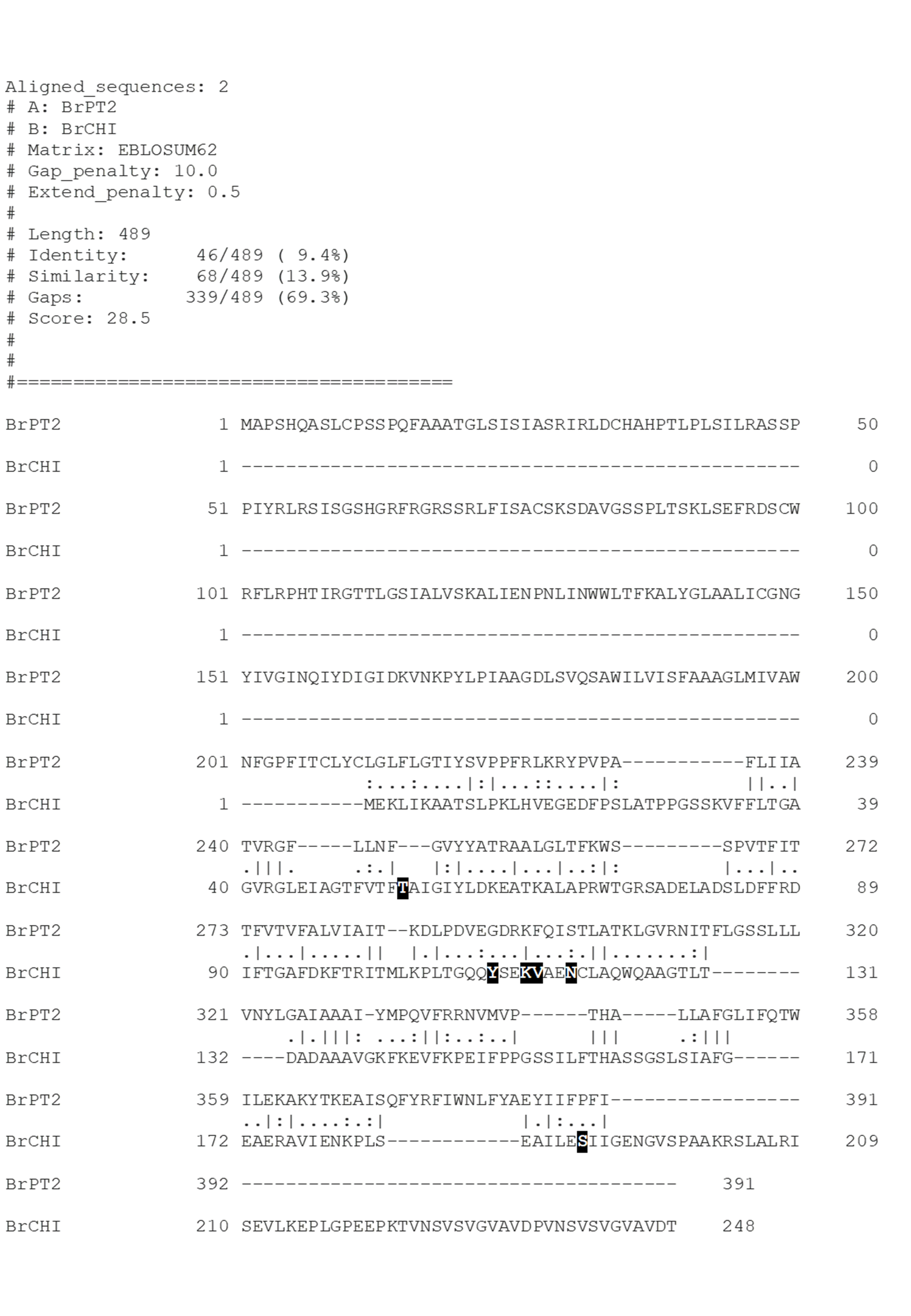

Supplement: Supplemental Information 2 — Conserved active sides of chalcone isomerase are shown in white on black background. [file peerj-08-9094-s002.png]

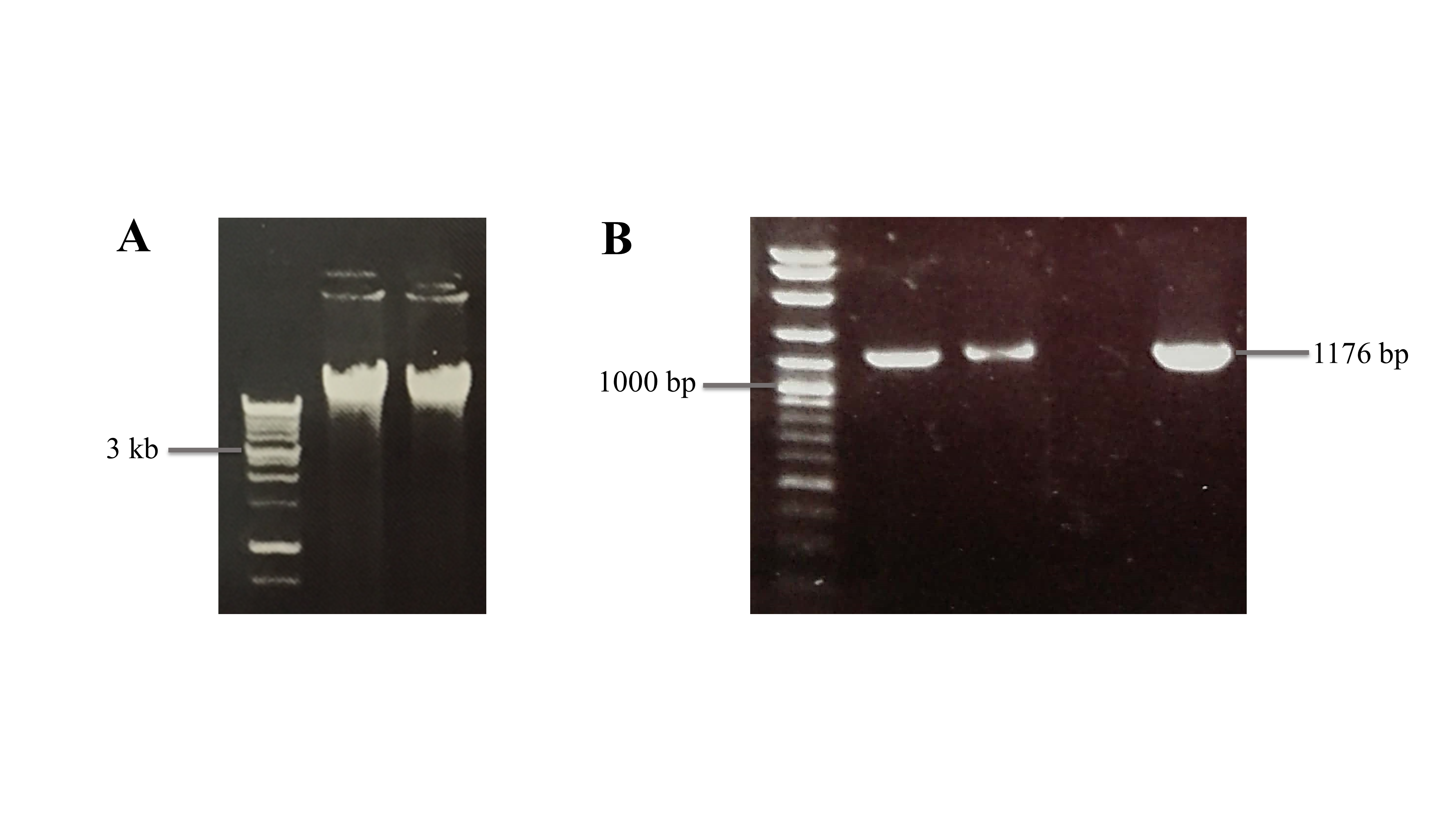

Supplement: Supplemental Information 3 — M: DNA marker; Lanes 1 and 2: genomic DNA of transformed cells. Lane M: molecular weight marker, Lanes 1 and 2: single band at ~1.2 KB was detected in positive transformants, Lane 3: wild type, and Lane 4: positive control. [file peerj-08-9094-s003.png]

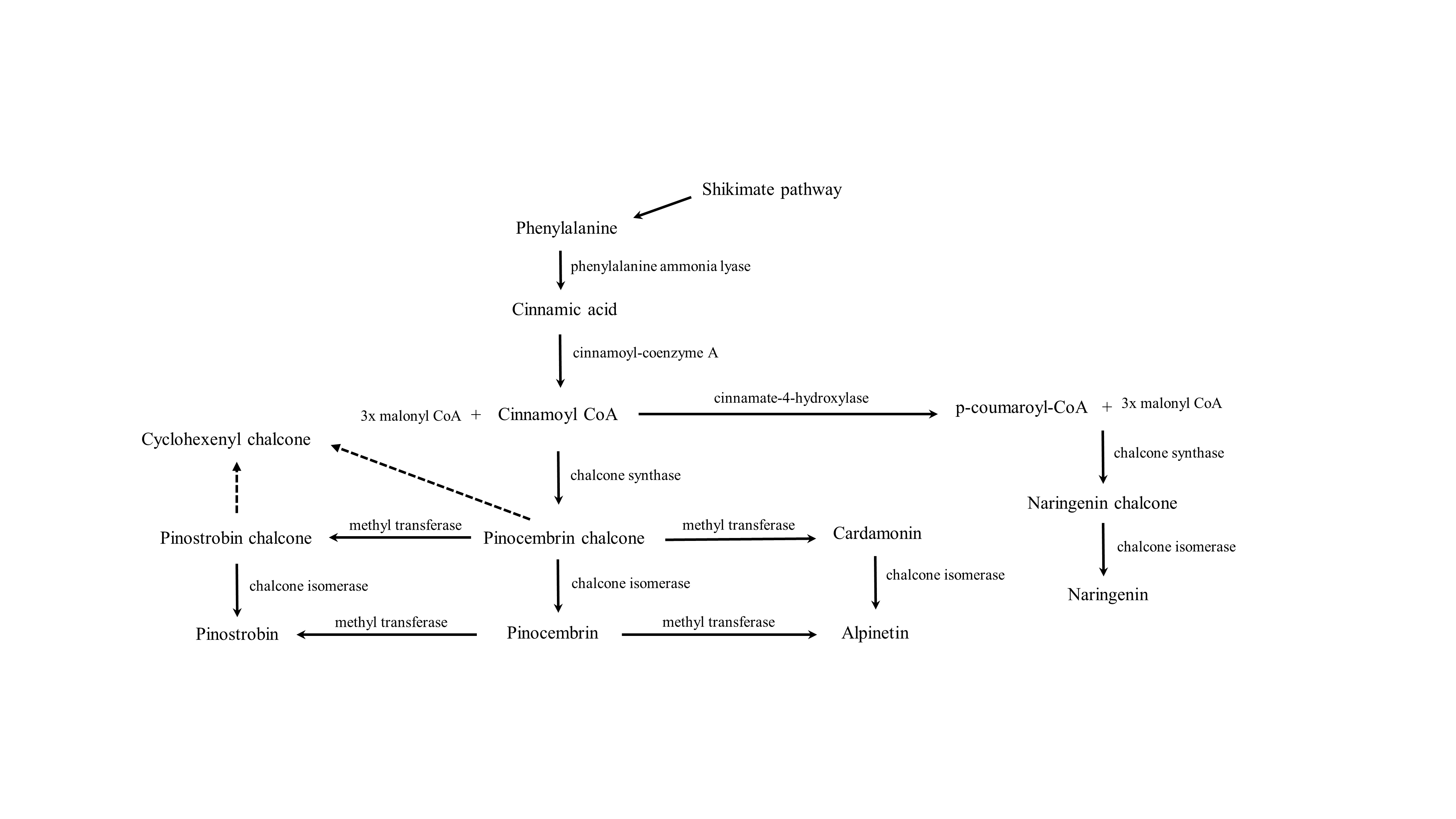

Supplement: Supplemental Information 4 [file peerj-08-9094-s004.png]
