# Supplementary material for: Enhancing flavonoid production by promiscuous activity of prenyltransferase, BrPT2 from Boesenbergia rotunda
Source: PeerJ. 2020 May 1;8:e9094. doi: 10.7717/peerj.9094 (PMC7197402; doi:10.7717/peerj.9094)
Supplement: Supplemental Information 7 [file peerj-08-9094-s007.docx]

>BrPT2

ATGGCTCCTTCTCACCAAGCTTCTCTCTGTCCTTCCAGCCCTCAGTTTGCTGCCGCCACCGGCCTTTCCATCTCCATTGCCTCCCGCATTCGTCTCGACTGCCATGCGCACCCAACCCTCCCTCTCTCCATCCTTCGAGCCTCCTCGCCGCCGATCTATCGCCTACGCTCCATTTCTGGCTCTCATGGACGTTTCCGAGGAAGATCCTCGCGTTTGTTCATCTCGGCATGTTCTAAGTCTGATGCTGTTGGTTCCAGCCCATTAACCTCAAAATTATCAGAGTTCAGAGATTCATGCTGGAGATTCCTAAGACCACACACAATCCGAGGAACTACATTGGGTTCCATAGCTTTAGTTTCAAAAGCTTTGATAGAGAATCCAAATCTTATAAATTGGTGGTTGACATTCAAGGCATTATATGGGCTTGCAGCTCTTATATGTGGCAATGGTTACATAGTTGGGATCAATCAGATTTATGACATCGGTATTGACAAAGTGAACAAGCCATATTTGCCTATAGCTGCTGGAGATCTATCTGTTCAGTCAGCTTGGATTTTAGTGATCTCTTTTGCAGCAGCTGGCCTCATGATTGTTGCATGGAATTTTGGTCCATTCATTACTTGTCTTTATTGTTTGGGACTGTTTCTAGGCACTATATATTCTGTTCCTCCATTTAGATTGAAGAGATATCCTGTTCCAGCATTTCTTATAATTGCAACGGTACGTGGGTTTCTTCTCAATTTTGGTGTATATTATGCTACTCGAGCTGCATTGGGCCTAACTTTCAAGTGGAGTTCACCTGTAACTTTCATCACAACATTCGTGACAGTATTTGCTTTGGTCATTGCTATAACTAAAGATCTTCCGGATGTTGAGGGCGACCGCAAGTTTCAGATTTCAACCTTGGCAACAAAGCTTGGTGTTAGGAACATCACATTCCTTGGTTCAAGTCTTCTATTGGTGAATTACCTTGGTGCTATTGCTGCAGCAATTTATATGCCACAGGTCTTTAGAAGGAACGTAATGGTGCCAACACATGCTTTGCTTGCATTCGGATTGATTTTCCAGACATGGATTTTGGAAAAAGCAAAATACACCAAGGAGGCTATATCTCAGTTCTATCGGTTTATTTGGAATCTTTTCTATGCCGAATACATTATTTTTCCTTTCATATAG
